# Navigated Percutaneous Sacroiliac Screw Fixation in Unstable Pelvic Ring Fracture

**DOI:** 10.7759/cureus.29897

**Published:** 2022-10-04

**Authors:** Shafiq Azhar, Syed Syafiq, Nik Kamarul Arif, Ahmad Faiz Mohamed Khalil, Mohd Hisam Muhamad Ariffin

**Affiliations:** 1 Orthopaedics and Traumatology, Hospital Canselor Tuanku Muhriz UKM, Kuala Lumpur, MYS; 2 Orthopedics and Traumatology, Hospital Canselor Tuanku Muhriz/National University of Malaysia, Kuala Lumpur, MYS; 3 Orthopaedics, Hospital Canselor Tuanku Muhriz UKM, Kuala Lumpur, MYS; 4 Orthopaedics and Traumatology, Universiti Kebangsaan Malaysia Medical Centre, Kuala Lumpur, MYS; 5 Spine Surgery, Universiti Kebangsaan Malaysia Medical Centre, Kuala Lumpur, MYS

**Keywords:** trauma and orthopedic surgery, damage control resuscitation, general trauma surgery, surgical navigation system, pelvic ring injury

## Abstract

Since the late 1990s, navigation systems have been widely used in a variety of orthopaedic surgical procedures, with the majority of these procedures being complex arthroplasty surgeries and the correction of spinal abnormalities. Navigation systems are, however, infrequently used in trauma cases, especially in unstable pelvic ring fractures. The conventional method of percutaneous sacroiliac screw fixation typically used fluoroscopic image intensifiers to fix unstable pelvic ring fractures. We will examine how navigation systems can be used in trauma situations, particularly those involving unstable posterior pelvic ring fractures and focus on the advantages and disadvantages that we experienced during management.

## Introduction

Nearly 3% of all skeletal fractures are pelvic [1]. The death rate following an untreated unstable pelvic fracture could reach as high as forty percent as a result of presacral venous plexus haemorrhage [2]. In cases of acute trauma, pelvic external fixators are a popular stabilisation procedure used to minimise damage.

However, in cases of haemodynamically stable patients, open reduction and internal fixation is always a treatment of choice as it provides proper anatomical reduction and biomechanically more stable fixation, thus providing early mobilisation for the patient [3].

The navigation system was first introduced in the mid-1990s, whereby most of its uses were in neurosurgical surgery [3]. Recently, it has gained momentum and is being utilised in orthopaedic surgery, commonly in spinal surgery or arthroplasty surgery [4]. However, the prevalence of using CT-guided navigation systems in trauma cases is still low and not widely reported. In this study, we will be sharing our experience in managing two unstable pelvic ring fracture patients using the navigation system.

## Case presentation

Two unstable pelvic ring fractures were brought to our emergency department as a result of trauma between January 2021 and June 2021. In the first example, the patient experienced symphysis pubis disruption together with left sacral ala longitudinal fracture through the neuroforamen which is classified as Denis Zone 2. Meanwhile, the second patient experienced a right sacral ala fracture with an ipsilateral superior and inferior pubic rami fracture. The advanced trauma team at Hospital Canselor Tuanku Muhriz (HCTM) used a navigation system which lent by Medtronic Malaysia Sdn Bhd to perform surgery on both pelvic cases.

Case 1

A 42-year-old male presented to HCTM following a motor vehicle accident. Upon arrival at the emergency department, the primary survey was conducted and the patient had tenderness over his left hip area and pelvis spring tested positive on the same side. His blood pressure was noted to be hypotensive, and his heart rate was tachycardic within one hour of presentation. A pelvic binder was immediately applied, and resuscitation protocol was initiated as per Advanced Trauma Life Support (ATLS) guidelines by the Emergency Department team.

**Figure 1:**
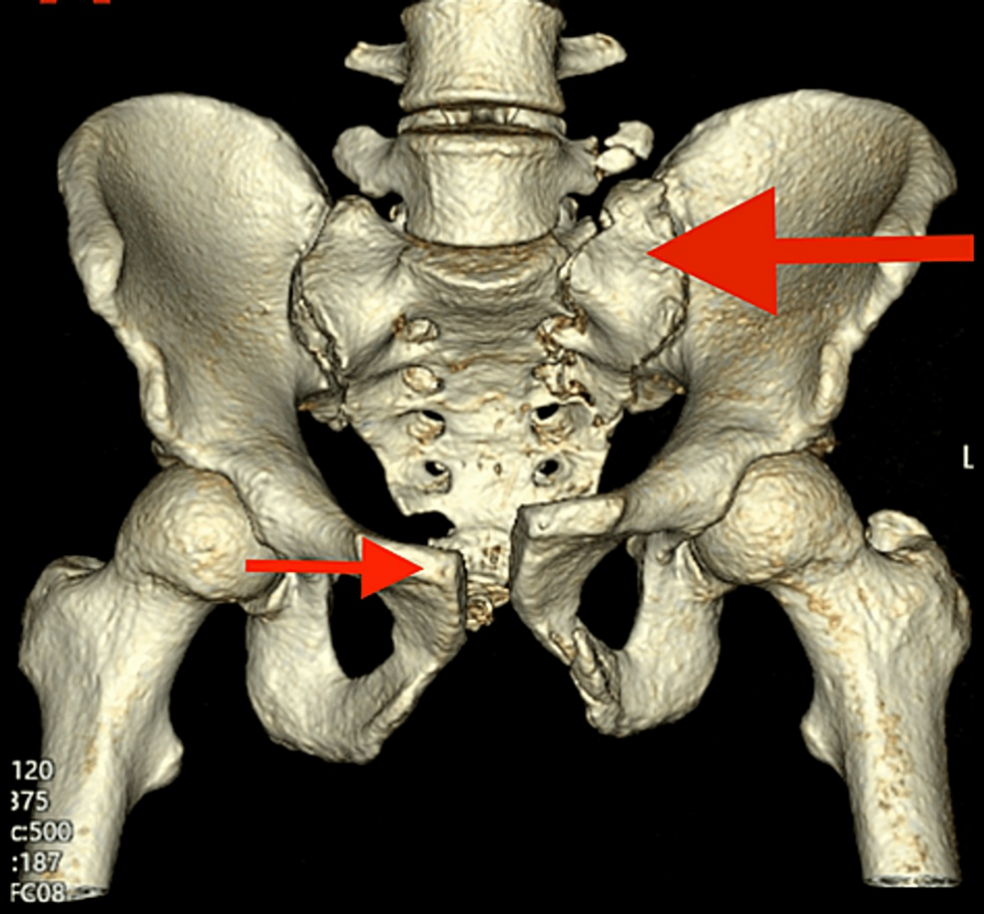
CT scan of the first patient showing symphysis pubis diastasis with left sacral ala fracture.

The patient underwent basic trauma survey radiographs which include cervical, chest and pelvis radiographs. From the radiographs, we noted a widening of the symphysis pubis and left sacral ala fracture. Since it was a high-impact injury, we proceeded with a CT scan of the pelvis to help us determine whether there was any organ or intraabdominal injury. From the CT scan, we may also be able to see fracture configuration detail or any intraarticular involvement which is important for preoperative planning. CT reported that the patient sustained a closed left sacral ala fracture with intra-articular extension into the adjacent left sacroiliac joint of the left ilium as well as a widening of the pubis symphysis measuring more than 2 cm of disruption (Figure 1).

Case 2

A 28-year-old male presented to HCTM following a fall from a 10-storey height while working. Upon presentation to the hospital, the patient was immediately intubated due to poor GCS. A complete primary survey was performed and noted that there was a positive pelvic spring test over the right side.

**Figure 2:**
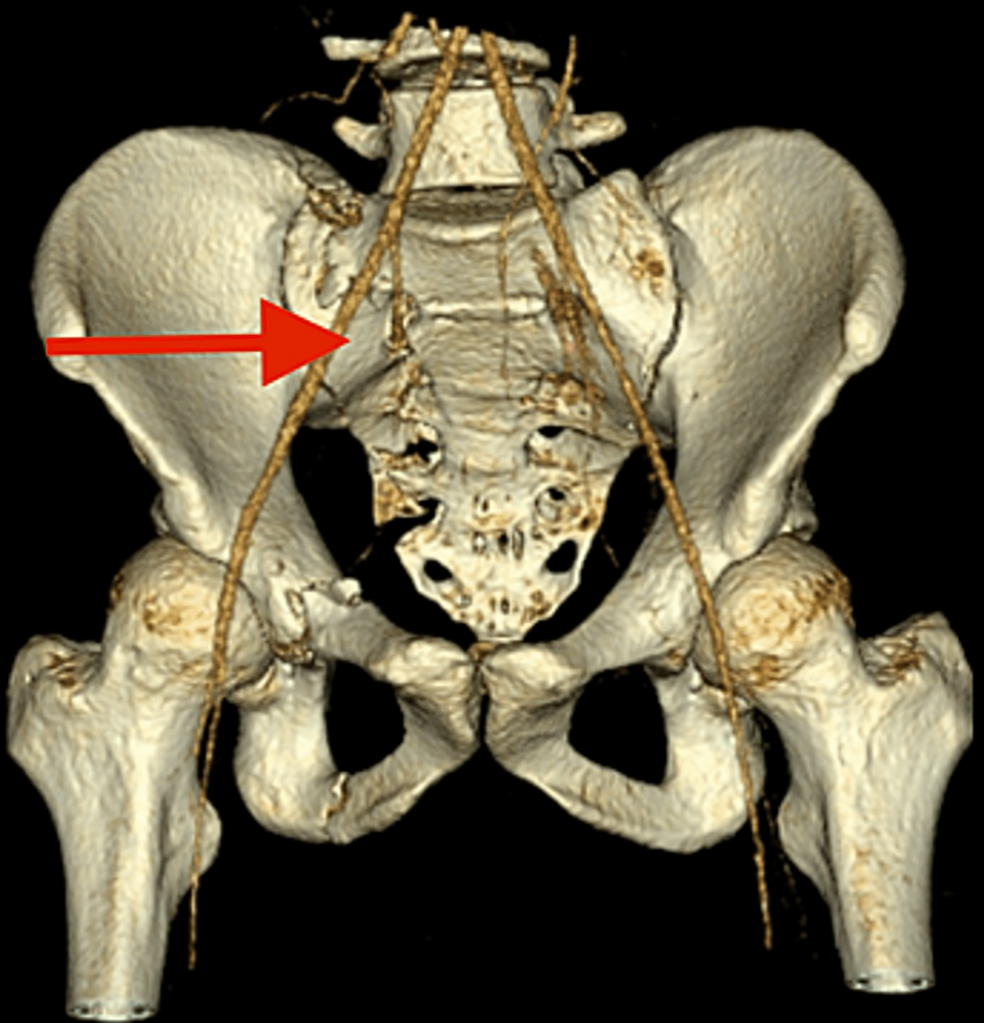
CT scan of the second patient showing right sacral ala fracture with ipisilateral superior and inferior pubic rami fracture.

CT pelvis was performed as part of adjunct to the primary survey. CT reported that the patient sustained a right sacral ala longitudinal fracture with ipsilateral superior and inferior pubic rami fracture (Figure 2).

For the first patient, unstable pelvic ring injury was temporarily stabilised using pelvic external fixators. Over the course of five days, the patient's condition was stabilised and a meticulous strategy was implemented to permanently fix the unstable pelvic ring injuries. We decided to use one 3.5mm pubic symphysis locking plate (Synthes) for the symphysis pubis diastasis and two 6.5mm cannulated half-threaded cancellous screws with a washer (Synthes) for the sacral ala fracture. Meanwhile, for the second patient, two 6.5mm cannulated half-threaded cancellous screws with a washer (Synthes) were used for the sacral ala fracture. The operation was done on the second day of the trauma.

**Figure 3:**
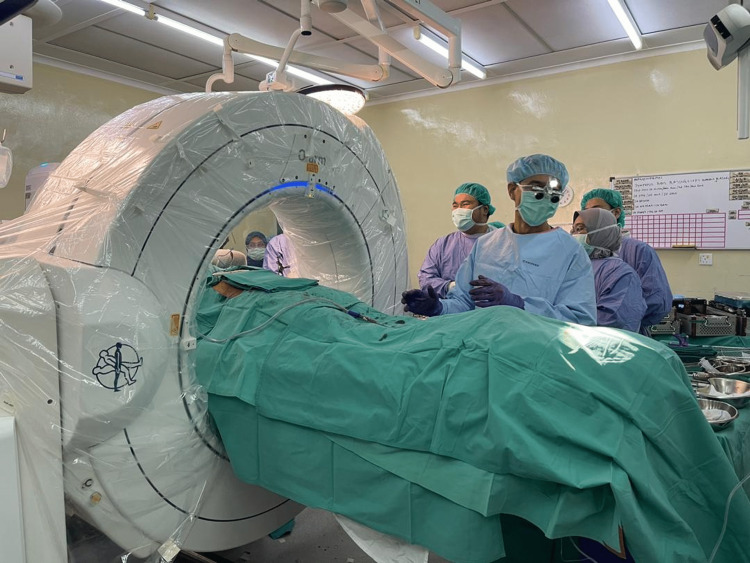
The O-arm machine placed isocentrically to the patient.

Both operations were done under general anaesthesia with the patient placed on a Jackson table in a supine position. The navigation system, Stealth Station (Medtronic), was connected to the O-arm machine (Medtronic). The machine was positioned isocentrically over the patient. A reference probe was fixed to the anterior superior iliac spine of the unaffected pelvis to serve as a signal receiver (Figure 3).

To confirm the fracture site and position, first anteroposterior and lateral 2D radiographs were taken. Then 3D computerised tomography scan imaging was performed with all the medical personnel leaving the operating theatre. A 3D reconstruction image of the pelvis was generated and integrated together with the previous radiographs onto the Stealth Station screen to provide real-time imaging throughout the surgery.

**Figure 4:**
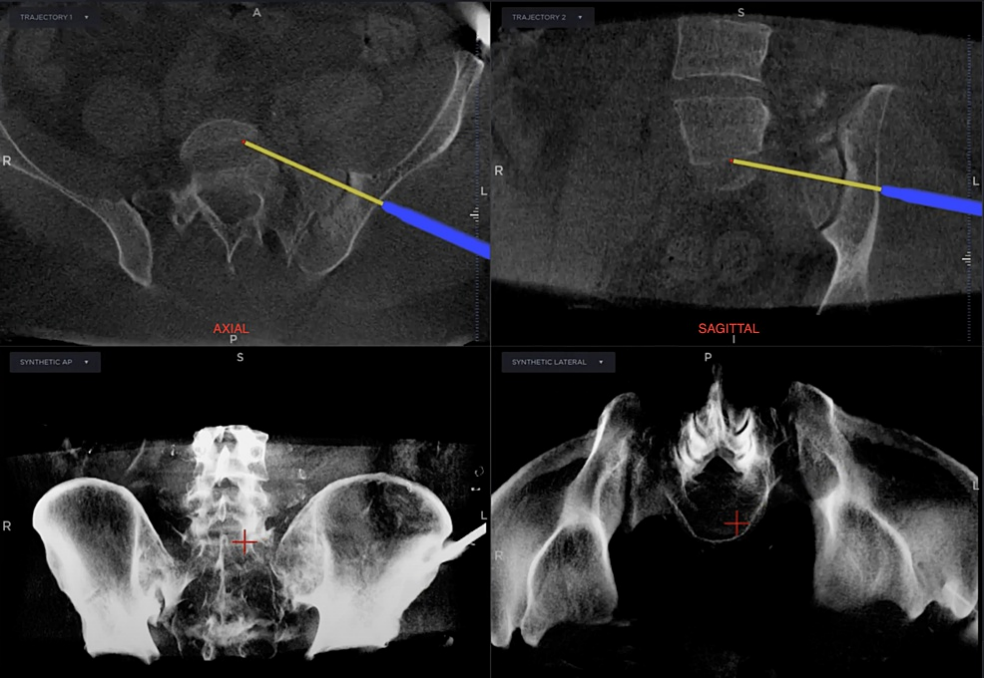
Live 3D images in the axial and sagittal planes from the Stealth Station screen showing the screw trajectory for the first patient.

After the image had been generated, we proceeded with screw fixation of the sacroiliac joint. A stab Incision was made over the lateral aspect of the hip and a 3.2mm guidewire wire passed perpendicular to the sacroiliac joint under navigation guidance. Two cannulated half-threaded 6.5mm screws with a washer inserted perpendicular to the sacroiliac joint. Screw placement was confirmed intraoperatively in all planes including sagittal, coronal and transverse using the navigation system (Figure 4).

**Figure 5:**
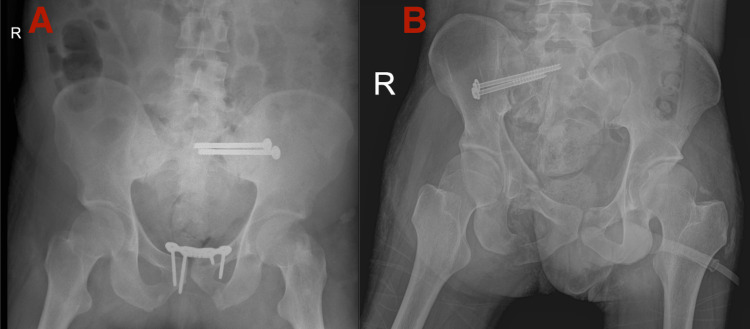
Postoperative radiograph showing left sacral ala screw fixation and reduced symphysis pubis disruption for the first patient (A) and right sacroiliac screw fixation for second patient (B) following osteosynthesis.

The total operation duration for the first patient was 1 hour and 45 minutes while it took 1 hour and 20 minutes for the second patient. Repeated pelvic radiographs on day 1 post-operation showed an acceptable reduction with the implant in situ (Figures 5A, 5B). The patient recovered well with no neurological deficit and was discharged on day 3 postoperatively.

## Discussion

The posterior sacroiliac complex, which is made up of the sacroiliac, sacrotuberous, and sacrospinous ligaments, is essential for maintaining pelvic stability. A fracture is deemed to be unstable if there is any damage to the posterior sacroiliac complex. According to the Tile classification, both of the cases were categorised as type B [5].

The technology that we used for both surgeries is the O-arm machine and Stealth Station navigation system from Medtronic. It provides a combination of 3D surgical imaging with real-time navigation during the surgery. The navigation system in general provides us with better-intended trajectory aids and accuracy in implant placement intraoperatively for unstable posterior pelvic ring fracture [3,6,7]. The images generated by the machine were 3D, high-resolution live images of the screw as well as the bone. The system provides images in an anteroposterior, axial and coronal plane view of the bone and screw on the screen as compared to the conventional C-arm machine, which only provides images in a 2D plane.

It is believed that the incidence of sacroiliac screw malposition rates was as low as 0.1% when using a navigation system as compared to a conventional C-arm, which accounts for 2.6% [7]. Therefore, in both of our cases, the proper length and diameter of the screw along with its trajectory can be easily visualised on the screen [3,7]. Moreover, with the help of these live images, any risk of perforation of the sacral neuroforamina and sacral cortices can be avoided [7].

Additionally, using navigation is advantageous for us for the first case because adequate visualisation can be achieved even when the patient is thought to have a higher body mass index (BMI). On the other hand, proper iliosacral screw placement for obese patients is sometimes challenging and time-consuming when using C-arm [7].

Other than that, we were able to minimise the correction during the drilling of the guidewire process, which may reduce the loss of bone stock. Any loss of bone stock may cause improper screw placement due to loosening of the screw tract [6,7]. The technology also allows us to change screws that appear to be mispositioned on the screen in order to achieve optimal fixation.

However, navigation systems in general take a longer setup time [3,6,7]. The machine needs to be properly positioned by the staff who are familiar with the machine in order not to interfere with the surgical field and at the same time to allow the reference probe to receive the signal properly. Any intraoperative interference of the signal may also contribute to the delay in operation time. In our example, the total setup time took approximately 30 minutes for the first patient and 20 minutes for the second patient to complete. However, once the whole process of merging the image is done, fixation of the pelvis takes a significantly shorter time to complete [3,6,7]. For instance, screw placement for the first patient only took 1 hour and 15 minutes to complete, meanwhile, the second patient took 1 hour to complete.

Utilisation of the navigation system provides less radiation to the surgeon as compared to the conventional C-arm machine. The radiation exposure is only during the initial phase of the CT scan, which also requires all the staff to be evacuated from the operation room during scanning. Once images are generated on the screen, no more radiation is needed during the whole process intraoperatively [3,7]. However, the radiation level in the patient would be significantly higher compared to the regular C-arm machine [7].

## Conclusions

Computer-assisted navigation devices can help pelvic surgeons to place screws more accurately in unstable posterior pelvic ring fractures, which can help lower patient morbidity. This has been demonstrated by the good postoperative outcome, no neurological deficit, and the absence of the necessity for revision surgery.

However, the whole surgery may take a longer time due to the startup and proper positioning of the machines. With proper training and exposure, these shortcomings can be improved, and we can provide the best treatment for the patients.

In conclusion, a navigation system will benefit in trauma cases and can be used to enhance surgical methods and achieve better results. The future lies in this technical innovation, and we intend to explore this technology more at our centre.
